# PanPA: generation and alignment of panproteome graphs

**DOI:** 10.1093/bioadv/vbad167

**Published:** 2023-11-24

**Authors:** Fawaz Dabbaghie, Sanjay K Srikakulam, Tobias Marschall, Olga V Kalinina

**Affiliations:** Institute for Medical Biometry and Bioinformatics, Medical Faculty and University Hospital Düsseldorf, Heinrich Heine University Düsseldorf, 40225 Düsseldorf, Germany; Center for Digital Medicine, Heinrich Heine University, 40225 Düsseldorf, Germany; Helmholtz Institute for Pharmaceutical Research Saarland (HIPS), Helmholtz Center for Infection Research (HZI), Saarbrücken, Germany; Helmholtz Institute for Pharmaceutical Research Saarland (HIPS), Helmholtz Center for Infection Research (HZI), Saarbrücken, Germany; Graduate School of Computer Science, Saarland University, 66123 Saarbrücken, Germany; Interdisciplinary Graduate School of Natural Product Research, Saarland University, 66123 Saarbrücken, Germany; Institute for Medical Biometry and Bioinformatics, Medical Faculty and University Hospital Düsseldorf, Heinrich Heine University Düsseldorf, 40225 Düsseldorf, Germany; Center for Digital Medicine, Heinrich Heine University, 40225 Düsseldorf, Germany; Helmholtz Institute for Pharmaceutical Research Saarland (HIPS), Helmholtz Center for Infection Research (HZI), Saarbrücken, Germany; Drug Bioinformatics, Medical Faculty, Saarland University, 66421 Homburg, Germany; Center for Bioinformatics, Saarland University, 66123 Saarbrücken, Germany

## Abstract

**Motivation:**

Compared to eukaryotes, prokaryote genomes are more diverse through different mechanisms, including a higher mutation rate and horizontal gene transfer. Therefore, using a linear representative reference can cause a reference bias. Graph-based pangenome methods have been developed to tackle this problem. However, comparisons in DNA space are still challenging due to this high diversity. In contrast, amino acid sequences have higher similarity due to evolutionary constraints, whereby a single amino acid may be encoded by several synonymous codons. Coding regions cover the majority of the genome in prokaryotes. Thus, panproteomes present an attractive alternative leveraging the higher sequence similarity while not losing much of the genome in non-coding regions.

**Results:**

We present PanPA, a method that takes a set of multiple sequence alignments of protein sequences, indexes them, and builds a graph for each multiple sequence alignment. In the querying step, it can align DNA or amino acid sequences back to these graphs. We first showcase that PanPA generates correct alignments on a panproteome from 1350 *Escherichia coli*. To demonstrate that panproteomes allow comparisons at longer phylogenetic distances, we compare DNA and protein alignments from 1073 *Salmonella enterica* assemblies against *E.coli* reference genome, pangenome, and panproteome using BWA, GraphAligner, and PanPA, respectively; with PanPA aligning around 22% more sequences. We also aligned a DNA short-reads whole genome sequencing (WGS) sample from *S.enterica* against the *E.coli* reference with BWA and the panproteome with PanPA, where PanPA was able to find alignment for 68% of the reads compared to 5% with BWA.

**Availalability and implementation:**

PanPA is available at https://github.com/fawaz-dabbaghieh/PanPA.

## 1 Introduction 

Prokaryotes have been living on Earth for billions of years, during which they continued to evolve rapidly. With the geochemical changes on the planet, bacteria needed to adapt in order to survive these environmental and habitat changes, which led to their vast genetic diversity (Dunlap 2001). Looking at stable environments like garden soil, lakes, or coastal seawater that do not experience extreme environmental changes, we observe a large diversity of prokaryotic organisms; and it is expected that not more than 1% of the bacteria in these samples can be cultivated in the lab (Amann *et al.* 1995), which suggests that the true diversity is even larger. It has been estimated that the total number of prokaryotic cells on Earth is around $4-6\times 10^{30}$ and their cellular carbon amount is $3.5-5.5\times 10^{14}$ kg (Whitman *et al.* 1998).

With the fast development of sequencing technologies, and, as a consequence, the fast production of large amounts of sequences, diversity and variability of prokaryotic genomes has become even more apparent (Perna *et al.* 2001). One way to understand new genomes and their diversity is by comparing their DNA to some well-studied reference genomes of the same species. Therefore, sequence alignment has been a cornerstone in bioinformatics for many years: it is extremely useful for finding homology between genes and proteins, identifying conserved regions, understanding evolutionary relationships between organisms, and many other important tasks (Higgins 2001).

In many cases, sequencing reads of a new sample are directly analyzed by comparing them to a reference genome, i.e. to one genome representative of the species. However, the linearity of a reference genome can lead to biases, e.g. if the query sequence contains a non-reference allele, which leads to incorrect or missing alignments (Chen *et al.* 2021). These effects are more pronounced in highly variable organisms like bacteria. To describe this genomic variability, the terms “core” and “accessory” genes were first coined by Tettelin *et al.* (2005), where the “core” genes refer to essential genes (e.g. housekeeping genes) that are found in all or nearly all isolates, and the “accessory” genes (sometimes called “dispensible” genes) refers to the genes that are not present in every genome or isolate sequenced. The term “pangenome” was first introduced by Sigaux (2000) describing a database of tumor genome and transcriptome alterations, as well as relevant normal cells. In bacteria, pangenome refers to all core and accessory genes observed in a species.

In recent years, graph representations of pangenomes have become more widespread, providing a more complete picture of pangenomes than a simple distinction into core and accessory genes. In graph-based models of pangenomes, one represents the genomic variability of a population using a graph data structure where nodes are labeled with sequences and edges connect nodes representing sequences that are adjacent to each other in one or more genomes in a population (Eizenga *et al.* 2020). One can then use these graph data structures as a reference instead of using a linear reference to reduce reference biases (Paten *et al.* 2017), which entails the need to align sequences to a graph.

Sequence alignment and pattern matching to a string graph is not a new problem; it has been described almost three decades ago. Pioneering studies include Manber and Wu (1993), where pattern matching on hypertext was described and Akutsu (1993), where an algorithm for exact pattern matching to a hypertext in a tree structure was developed. In 1995, Park and Kim (1995) described regular pattern matching on a directed acyclic graph (DAG). Later on, an algorithm that does pattern matching on “any” hypertext was developed by Amir *et al.* (2000), then Navarro (2000) improved both time complexity and space complexity.

In 2002, these algorithms were adopted for biological data by Lee *et al.* (2002), where the Partial-Order Alignment algorithm was used for generating an MSA in a graph representation, the algorithm allows the alignment of a sequence against this graph representation. In essence, it is a modified version of the common sequence alignment with dynamic programming (DP) algorithms, where all the incoming edges connecting a certain node in the graph to other nodes are considered while calculating the cell’s score to find the best path of the sequence through the graph. In recent years, several tools have been introduced to perform sequence-to-graph alignments with better speeds and accuracy (Ivanov *et al.* 2020, Rautiainen and Marschall 2020, Sirén *et al.* 2021).

So far, all tools for pangenomes have mostly been implemented to be used for different samples or strains in a single species: in bacteria, e.g. for *Escherichia coli* (Colquhoun *et al.* 2021), in plants, for *Cucumis sativus* (Li *et al.* 2022), and in humans (Eizenga *et al.* 2020, Li *et al.* 2020), including the work of the Human Pangenome Reference Consortium (Liao *et al.* 2023). Due to the high diversity in bacteria, these tools typically cannot be used for inter-species comparisons at the DNA level, as the diversity is too high to make meaningful alignments. This problem is even more exacerbated in highly diverse and less-studied clades, e.g. *Actinomycetes* or *Myxobacteria*, which are an important source of natural products that can be used in drug discovery (Gerth *et al.* 2003). The diversity in these clades is much higher than what is already described due to limitations in cultivation and in-lab growth (Mohr 2018).

In these cases, however, one can still trace the sequence similarity by switching to amino acid alignments, i.e. looking only into coding regions, as these alignments will have a higher quality compared to DNA sequence alignments, due to several reasons. First, amino acid sequences are evolutionary more conserved compared to the total genome DNA sequence, as proteins have a specific biological function. Moreover, as the amino acid alphabet is larger, the “signal-to-noise ratio” is better (Wernersson and Pedersen 2003). The same amino acid can be encoded by several codons, hence, a part of mutations in DNA are not visible on the amino acid level. Second, some of the errors introduced during sequencing can cause a frameshift during alignments, which can be avoided when using amino acids (Sheetlin *et al.* 2014). Third, in amino acid sequence alignment, we usually use a substitution matrix instead of just edit distance in DNA sequence alignment, better capturing biological reality (Bininda-Emonds 2005). In prokaryotes, the fraction of non-coding regions in the genome can range from 5% to 50%. However, for the vast majority, the fraction is <18% (Rogozin *et al.* 2002), further motivating a focus on coding sequences.

Here, we propose a new tool PanPA to conduct pangenomic analyses that considers amino acid, or protein sequences. PanPA allows building DAGs for each individual protein or protein cluster in order to represent a pangenome. Computing alignments in amino acid space can give a big advantage in terms of finding more sequence similarity and being able to align more phylogenetically distant organisms against each other while losing relatively little genome information. Westbrook *et al.* (2017) showcased how aligning in protein space introduces significant improvements in alignment accuracy and functional profiling in a metagenome scenario. The idea of having many graphs representing a pangenome instead of one large graph was presented in Colquhoun *et al.* (2021); in their tool Pandora, the authors define a pangenome as a collection of “local graphs” where each local graph represents some locations in the genome that can be pre-defined by the user. PanPA combines the two ideas of (i) having a pangenome consisting of many smaller graphs, where each graph represents a protein or a protein cluster, and (ii) working in amino acid space rather than nucleotide sequences to support pangenomic analyses over larger evolutionary distances. We call such a collection of graphs a “panproteome.” We showcase the utility of PanPA by performing alignments of proteins and raw short reads from *Salmonella enterica* assemblies against a *E.coli* panproteome.

## 2 Methods

The idea behind PanPA is that we aim to build a panproteome of a collection of protein sequences or protein clusters. In this definition of a panproteome, each protein or protein cluster is represented as a separate graph. Therefore, our pipeline starts from multiple sequence alignments (MSAs) provided as input, where each MSA represents one protein or cluster, and the pipeline goes through three major steps:

1. Building an index from the input MSAs.
2. Constructing a directed graph from each MSA.
3. Aligning query sequences to these constructed graphs with the help of the index constructed from these MSAs.

### 2.1 Building index from MSAs

For each sequence *N* of length *m*, we define a substring s=N[i,j], where 0≤i≤j≤m−1, as a substring of *N* starting at position *i* and ending at position *j* with the length of j−i+1. A *k*-mer from a string *N* is then defined as a substring of length *k*. We also define a function min(S) that takes the set $S=\{s_{1},s_{2},\ldots ,s_{w}\}$ of size *w* containing *w* equally lengthed strings and returns the lexicographically smallest string in this set; we call this function a “minimizer.”

To construct a “*k*-mer based index,” for every string *N*, the seeds extracted from that string form a set $S_{\mathrm{seeds}}$ comprising every consecutive *k*-mer from *N*. $S_{\mathrm{seeds}}=\{N[0,0+k-1],N[1,1+k-1],\ldots ,N[i,i+k-1]\};\forall i\in \{0,\ldots ,(m-k)\}$, where each string *N* of length *m* will contain (m−k+1) k-mers. A “minimizer-based index” was originally developed by Schleimer *et al.* (2003) and was first used in bioinformatics to reduce storage requirements for sequencing data by Roberts *et al.* (2004). In this approach, for each sequence *N*, instead of taking the set of all consecutive *k*-mers as seeds, we take the set $S_{\mathrm{seeds}}$ that contains the minimizer of every consecutive window of w k-mers, i.e. we take the smallest seed in a set of seeds for each consecutive window of seeds. $S_{\mathrm{seeds}}=\{\min(S_{0,w}),\min(S_{1,w}),\ldots ,\min(S_{i,w})\};\forall i\in \{0,\ldots ,(m-w-k+1)\}$, where $S_{i,w}$ is a set of *w* consecutive *k*-mers starting at position *i* in the string *N*.

In PanPA, both a *k*-mer-based and a minimizer-based index are implemented and can be used alternatively. In both cases, the index stores a key-value map, where the keys are a set of all *k*-mers or (w,k)-minimizers extracted from each sequence in the input MSAs, and the values are ordered lists of MSAs where that key was found, the ordering of the values is based on the number of times that key showed up in that certain MSA. More on the indexing detail is described in Section 3.

### 2.2 Generating a DAG from a MSA

For this step, we developed a simple algorithm to turn each MSA into a graph in the “graphical fragment assembly” (GFA) format, where each original sequence from the MSA is represented in the GFA file as a path. This algorithm runs in O(n×m) time, where *n* is the number of sequences in the MSA and *m* is the length of the alignment and has two steps: (i) generating the graph, and (ii) compacting the graph.

#### 2.2.1 Generating the graph

We define an alphabet *A* as the amino acid alphabet, and a matrix $M=(a_{i,j})\in {\left\{A\cup -\right\}}^{m\times n}$, each column in matrix *M* is a vector ${\left\{A\cup -\right\}}^{n}$ and each row is a vector ${\left\{A\cup -\right\}}^{m}$. In a nutshell, the algorithm loops through each column vector at position *j* where 0≤j≤m−1, and for each of these vectors, it constructs a new node $\mathrm{nod}e_{j}(c)$ for each unique character c∈A. Edges are then added between two nodes $\mathrm{nod}e_{j_{1}}(c_{1})\to \mathrm{nod}e_{j_{2}}(c_{2})$ (where $j_{1}<j_{2}$) if and only if the characters $c_{1}$ and $c_{2}$ were consecutive in one of the rows in matrix *M* after ignoring the character {−}.

**Figure 1.**
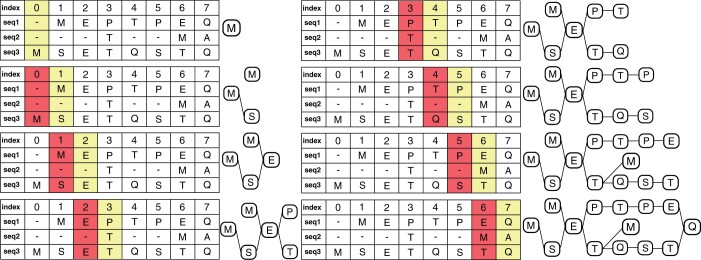
MSA to GFA: turning an MSA into a graph. The MSA in this example contains three sequences, - *MEPTPEQ*, - - - *T—MA*, and *MSETQSTQ*; and the step-by-step graph construction is shown on the panels from top to bottom. At every step, the yellow column is the current position and the red column is the previous one.

The algorithm is summarized in Algorithm 1. Consider an MSA with three sequences (Fig. 1); in this figure, the columns marked yellow are the “current” column in the loop, and the column in red is the “previous” column. The algorithm loops through the columns of the MSA, and at each column, it goes through each character in that column, if the character is new then a new node is initialized for this character (Lines 18–22 in Algorithm 1), otherwise, if the character is not new, i.e. a node was already constructed for that letter at that column, we assign the character a corresponding node identifier. After building nodes for a column *j*, i.e. the “current” column in the loop, we synchronize with column j−1, i.e. the “previous” column (if it exists) (Lines 2–10 in Algorithm 1), where we go through each row *i* in both columns, and for every row *i* we have three choices: (i) if $c_{i,j},c_{i,j+1}\in \{-\}$ (e.g. first two gaps in the second sequence in Fig. 1), then there is nothing to do; (ii) if $c_{i,j},c_{i,j+1}\in A$, then we need to draw an edge between $\mathrm{nod}e_{j}(c_{i,j})$ and $\mathrm{nod}e_{j+1}(c_{i,j+1})$; (iii) if $c_{i,j}\in A$ and $c_{i,j+1}\in \{-\}$ then we need to keep the character $c_{i,j}$ “saved” and continue going through the MSA until we reach a column j+x where $c_{i,j_{x}}\in A$ and x>1, then we can draw an edge between $\mathrm{nod}e_{j}(c_{i,j})$ and $\mathrm{nod}e_{j+x}(c_{i,j+x})$. An example of this final case in Fig. 1 is the second sequence, where Column 5 has a gap but Column 4 has a *T*; we keep track of this until we reach the character *M* in Column 7, where we construct a node for the character *M* in Column 7 and draw an edge between $\mathrm{nod}e_{5}(T)$ and $\mathrm{nod}e_{7}(M)$. Since we iterate through the MSA from left to right and draw edges between consecutive nodes, the resulting graph is directed and acyclic.

#### 2.2.2 Compacting the graph

Linear stretches of nodes can arise while generating a graph from an MSA. A set of consecutive nodes $\left\{\mathrm{nod}e_{j_{1}}(c_{1}),\mathrm{nod}e_{j_{2}}(c_{2}),\ldots ,\mathrm{nod}e_{j_{n}}(c_{n})\right\}$ is a linear stretch, if and only if each node in the set has an in-degree and out-degree of one, with an exception that the first node $\mathrm{nod}e_{j_{1}}(c_{1})$ can have a higher in-degree and the last node $\mathrm{nod}e_{j_{n}}(c_{n})$ can have a higher out-degree. Then, we can compact these nodes into one node and concatenate their sequences. For example, in Fig. 1 at the last step of constructing the graph, the stretch of nodes P→T→P→E can be compacted into one node.

PanPA’s final output is the compacted version of the graph in GFA format with each original sequence as a path entry in the output GFA.

Algorithm 1 Constructing a DAG from MSA

**Matrix** *M* {Matrix of dimensions m×n}

**Map** *nodes* {A map of node IDs: array of children IDs}

**Array** *previous* {Empty array of length *n*}

**Array** *current* {Empty array of length *n*}

**Int** *n* {Integer starting with 0}

1: **for**j∈{0…m}  **do**

2:  **for**i∈{0…n}  **do**

3:   **if**(current[i]≡None)&&(previous[i]≠None)**then**

4:    current[i]←previous[i]

5:   **else if**(current[i]≠None)&&(previous[i]≠None)**then**

6:    nodes[previous[i]].append(nodes[current[i]]))

7:   **else**

8:    pass

9:   **end if**

10:  **end for**

11:  previous←current

12:  **Array***current* {Empty array of length *m*}

13:  **Array**column←M[j] {characters in column j}

14:  **Map***seen* {empty map} {character: node ID}

15:  **for**i∈{0…m}**do**

16:   **if**column[i]∈seen**then**

17:    current[i]←seen[column[i]]

18:   **else**

19:    n←n+1

20:    nodes[n]←[]

21:    current[i]←n

22:    seen[column[i]]←n

23:   **end if**

24:  **end for**

25: **end for**

### 2.3 Aligning query sequences

#### 2.3.1 Amino acid queries

**Figure 2.**
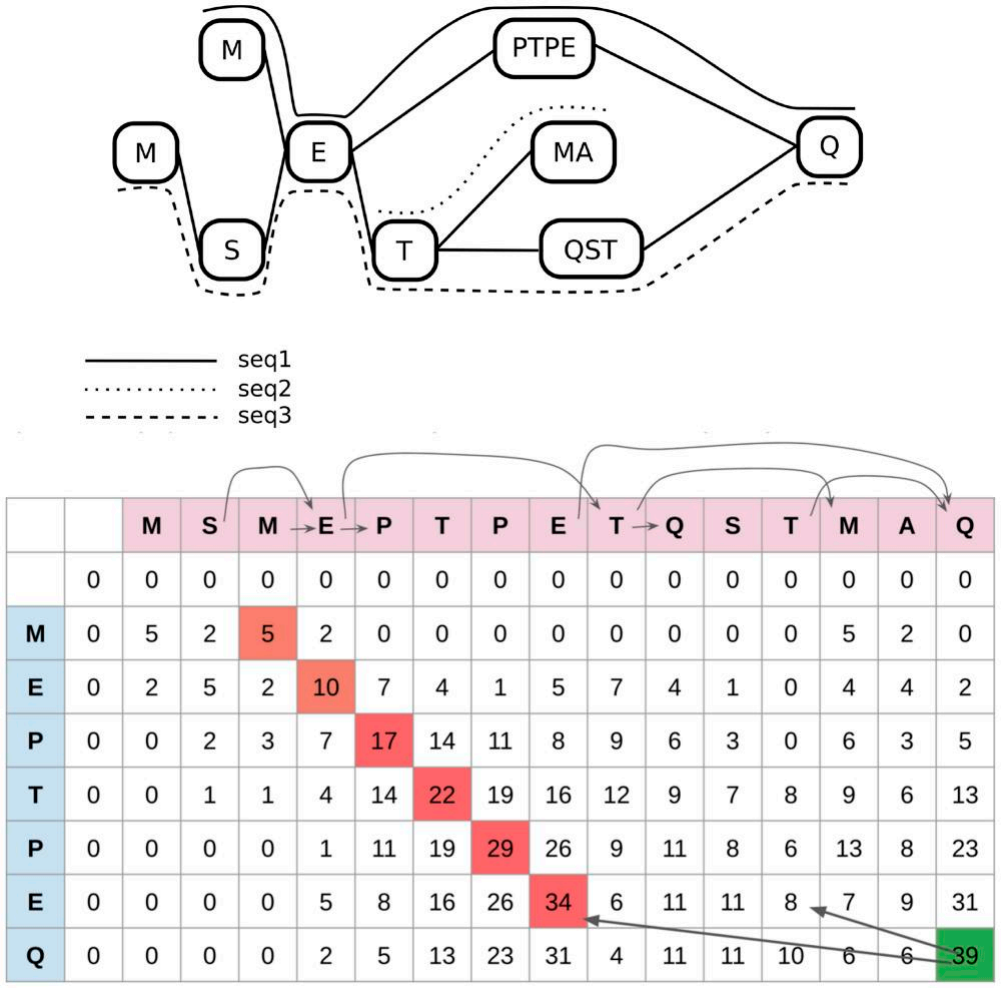
Alignment of a sequence to a protein graph. Top: example protein graph; bottom: the corresponding DP table. The ordered graph vertices are in the columns, and the query sequence is in the rows. Arrows between columns correspond to the graph edges. Arrows in the DP table correspond to potential previous cells in the DP process.

PanPA uses a modified version of the Smith–Waterman algorithm for local alignments (Smith and Waterman 1981) known as partial-order alignment (Lee *et al.* 2002). The main idea of the modification is that instead of looking at the previous character in the alignment to fill the DP table, we need to consider all incoming edges of a node. As each graph constructed from an MSA is a DAG, the graph can be topologically sorted generating a list of ordered vertices. The concatenation of the sequences of the ordered vertices is the target sequence to align against (Fig. 2).

The DP matrix is defined as $H=(a_{i,j})\in R^{\left(n+1)\times (m+1\right)}$, where *m* is the size of the query sequence *M*, and *n* is the size of the concatenated sequences *N* from the ordered vertices. We add one extra row and column filled with 0 as the initializing row and column. Similar to the Smith–Waterman algorithm, we need to fill cells of the DP table using the information from previous cells, considering the previous character. However, as some columns correspond to the first character of a node in the graph, we need to calculate the score of that cell based on all possible previous characters following all incoming edges to that node: for calculating the score of cell *i*, *j*, we take the max of all scores calculated considering all characters from the incoming edges $p_{l}$, where $p_{l}$ is the column index pointing to the previous character after following the incoming edge (1). To calculate a single score, we have three possible choices: a match/mismatch, an insertion, or a deletion (2).

(1) $$H_{i,j}=\underset{\forall l:p_{l}\in P_{in}}{\max}(\mathrm{score}(i,j,p_{l})).$$

(2) $$\mathrm{score}(i,j,p_{l})=\max\{\begin{array}{l} H_{i-1,p_{l}}+\mathrm{sub}(N[p_{l}-1],M[i-1])\\ H_{i-1,j}+\Delta \\ H_{i,p_{l}}+\Delta \\ 0 \end{array},$$

where δ is the gap score, and $\mathrm{sub}(c_{1},c_{2})$ is a function that takes two characters and returns the score based on a substitution matrix, e.g. Blosum62 (Henikoff and Henikoff 1992). Since our graphs are compacted, one node can have several characters. Therefore, if we are calculating the score for some $H_{i,j}$ and the column *j* does not correspond to the first character in the node, we can simply then use (2) with $p_{l}$ being simply j−1.

For tracing back the alignment, we use the same approach as in the classical Smith–Waterman algorithm, checking where the score of the cell came from to know which path our query sequence aligns to. For example, in Fig. 2, the last column corresponds to the character Q. When tracing back from it, we see two incoming edges: one leading to the character E and the other to the character T. The score is then calculated for each previous character and the maximum score is chosen, which corresponds to E at j=9. Hence, we continue the traceback from this cell. On the other hand, for j=9 there are no incoming edges, so we only need to look at j=8, and so on.

#### 2.3.2 Frameshift-aware DNA alignment

To align DNA sequences directly to amino acid graphs, while also accounting for insertion or deletion that could cause frameshifts, we used a method similar to Sheetlin *et al.* (2014), which considers frameshift-aware alignments of sequences, and adapted it to aligning to graphs. In our method, when filling the DP table, for each nucleotide in the query DNA sequence, we assume it is the third codon position nucleotide and consider it together with the previous two to form a codon. Supplementary Table S3 contains an example of a DP table for the frameshift-aware alignment. When filling a cell, we always look for the next (potential) amino acid to start three positions downstream in the DNA sequence and always make jumps across three rows.

To make this formulation frameshift-aware, we introduce two new types of diagonal jumps when calculating the score for a certain cell at (i,j):

1.
i−4,j−1 jump, which describes an insertion frameshift, when the DNA sequence has an extra nucleotide that introduced a frameshift, which moves the current alignment to the previous frame;
2.
i−2,j−1 jump, which describes a deletion frameshift, where the DNA sequence has one nucleotide deleted, which moves the current alignment to the next frame.

For the i−4,j−1 and i−2,j−1 jumps, we introduce a frameshift penalty σ.

Finally, the score for a cell in the DP table is calculated as:

(3) $$\mathrm{score}(i,j)=\max\{\begin{array}{l} H_{i-3,j-1}+\mathrm{sub}(\mathrm{trans}(N[i-2,i]),M[j-1])\\ H_{i-3,j}+\Delta \\ H_{i,j-1}+\Delta \\ H_{i-4,j-1}+\sigma \\ H_{i-2,j-1}+\sigma \\ 0 \end{array},$$

where *N* is the DNA sequence and *M* is the amino acid sequence, and the function trans() takes a codon and returns the equivalent amino acid, and the function sub() takes two amino acids and returns the substitution score between them.

## 3 Implementation

PanPA was built using Cython without any extra dependencies, where Cython was used mainly to optimize the core alignment algorithm. To facilitate the user, each step is implemented as a separate subcommand, which can be instrumental in finding optimal parameters for a certain dataset. The subcommands are build_index, build_gfa, and “align.”

**Figure 3.**
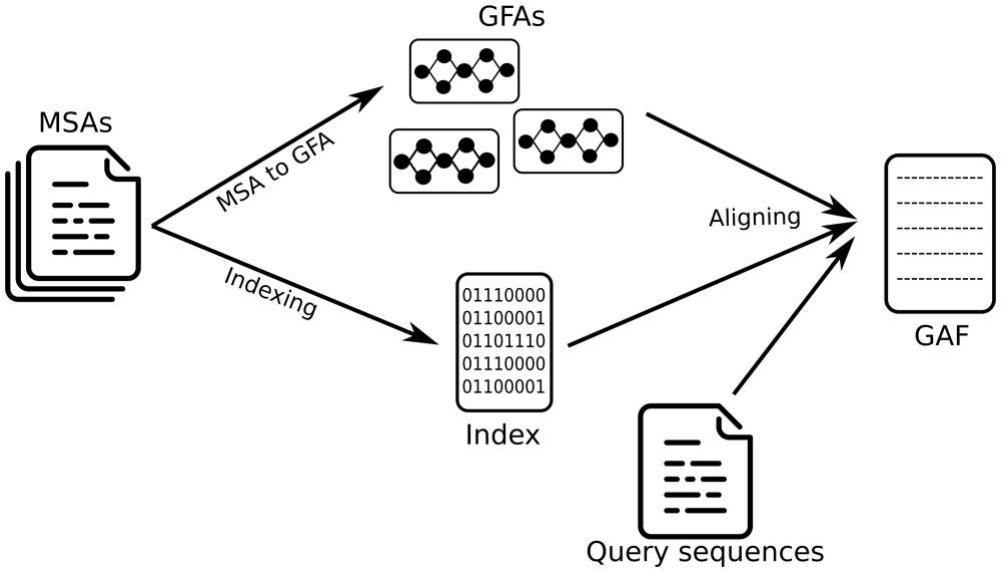
The general PanPA pipeline and its subcommands (in blue). Each subcommand can be also run separately or more than once with different parameters.

PanPA’s workflow proceeds in three key steps (Fig. 3). It starts with MSA files, where each MSA represents one protein or a protein cluster. This input is accepted by both build_index and build_gfa modules. The subcommand align takes a FASTA file with query sequences, the graphs, and the index file produced from the build_index step. PanPA then outputs the alignment in Graph Alignment Format files.

### 3.1 Indexing

In the indexing step, PanPA goes through each sequence in each MSA given and extracts the seeds from that sequence, be it *k*-mers or minimizers, depending on the user’s choice. Each seed is a key in a key-value map, and the value is a list of the MSA identifiers where that seed was found. In our implementation, the value vector is ordered based on the number of times that seed showed up in an MSA normalized for the number of sequences in that MSA. Therefore, the user can choose a cutoff limit on how many MSAs (equivalently, graphs) one seed can belong to, as some seeds can be promiscuous, especially a small value for *k* is used. Because the vector of hits is ordered, if the limit is an integer *n*, only the *n* top MSAs will be kept in the index.

For example, if we have three MSAs $m_{1}$, $m_{2}$, and $m_{3}$ containing 10, 7, and 3 sequences respectively, a seed $s_{1}$ is present in $m_{1}$ two times, in $m_{2}$ four times, and $m_{3}$ three times (with normalized counts being 0.2, 0.57, and 1, respectively), and the user cutoff is set to two, then in the resulting index, the seed $s_{1}$ points to a list $\left[m_{3},m_{2}\right]$.

In order to make extracting the minimizers from the consecutive windows faster, we used the Sliding Window Minimum algorithm (Carruthers-Smith 2011), which has a time complexity O(n), where *n* is the size of the input sequence.

### 3.2 Generating graphs

PanPA generates one DAG for each MSA and stores it in the GFA format. Therefore, when a seed in the index points to one MSA, we can align the query sequence to the graph that corresponds to that MSA. Moreover, because the original sequences in the MSA are encoded as paths with the path line in GFA, we cannot compact two adjacent nodes connected by one edge if not the same set of sequences pass through both these nodes. For example, consider an MSA with three sequences *MTQT*, - - *QT*, and *MT* - -. The corresponding graph has a linear stretch of four vertices (*M*, *T*, *Q*, and *T*) with one edge between every two consecutive vertices. However, if we compact all four vertices into one, we cannot write a path for Sequences 2 and 3 in the GFA file, because now they are contained inside this compacted node. Therefore, we can only compact nodes *M* and *T* together and nodes *Q* and *T* together; this way Sequence 3 is contained in the first node and Sequence 2 in the second (Supplementary Fig. S3).

### 3.3 Aligning

Given a query sequence, we count all the seed hits from the query to the MSAs using the index and generate a list of MSAs (equivalently, graphs) to align against. This list is sorted based on the number of hits: e.g. if the query sequence had five seeds, where four of them pointed to $m_{1}$, and one pointed to $m_{3}$, our list of matches will be $\left[m_{1},m_{3}\right]$. The user can also specify to how many potential MSAs/graphs can one query be aligned against, or choose to align to all matches. If, e.g. the limit of matches was set to one, our query sequence will only be aligned to $m_{1}$. Moreover, the user can specify a minimum acceptable alignment identity score, and only the alignments with scores equal or larger to this minimum threshold are returned. PanPA also uses a linear gap penalty and the user can choose one of many substitution matrices available.

## 4 Results

### 4.1 Validating PanPA on a panproteome of *E.coli*

We first wanted to validate that PanPA is able to find correct alignments. Therefore, we built a panproteome of *E.coli* and then realigned all sequences to it. To that end, we first downloaded 1351 *E.coli* assemblies that were marked as “Complete Genome” from RefSeq (O’Leary *et al.* 2016). We extracted every amino acid sequence corresponding to a coding region from the annotations provided in RefSeq and clustered them using mmseq2 (Hauser *et al.* 2016) with default parameters, resulting in 44 204 protein clusters. The distribution of the number of strains per cluster (Supplementary Fig. S1) has the characteristic U-like shape, which evidences the presence of the core genes that are present in nearly all assemblies (right part of the plot) and accessory genes that are mostly unique to one assembly or present in only a few (left side of the plot). Now that we had similar proteins clustered together, mafft (Katoh and Standley 2013) was used on each cluster to produce a corresponding MSA.

We then proceeded with PanPA to produce a DAG in GFA format from each MSA. We randomly selected 32 289 protein sequences from our MSAs collection. The random selection was done by, first, randomly selecting 10% of all the MSAs representing the protein clusters, then for each MSA chosen, we randomly selected 5% of sequences in that MSA. Therefore, we had a ground truth as to where each sequence comes from and to which graph it should align; and we expected that PanPA should align each of these sequences to the correct corresponding graph. We constructed a pipeline using Snakemake (Mölder *et al.* 2021) to run the indexing and alignments steps with a combination of several parameters to demonstrate the effect of different parameters on the correctness of the alignments.

**Figure 4.**
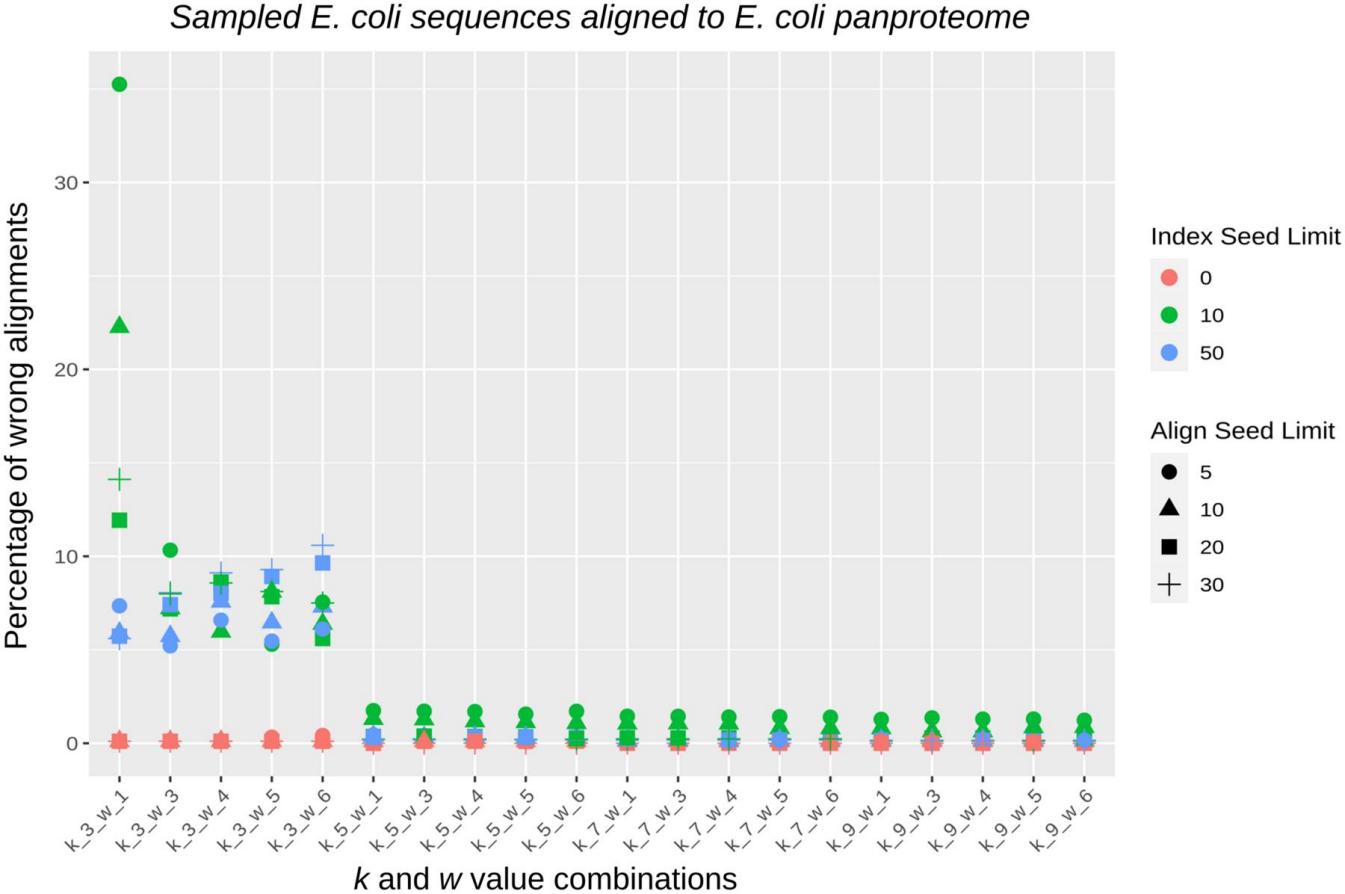
Effect of the different parameters on the fraction of wrongly aligned sequences, where a “wrong alignment” is a sequence being aligned to a different graph than the one it originated from. Each point is colored with respect to the seed hits limit (the limit of how many hits can each seed point to), and shapes correspond to the aligned hits limit (the limit of how many graphs can one sequence align to). For small *k* values, a high number of wrong alignments is produced, unless the index size is limited. The align seed limit has a relatively small effect on the percentage of wrong alignments.

We define a “wrong alignment” here when the highest-scoring alignment produced by PanPA corresponds to an alignment against a different graph/MSA than that where the sequence originated from. For k=3, we get a relatively high number of wrongly aligned sequences, unless the index stores all the seed hits (the value 0 in the figure, with the red marks), whereas higher *k* values produce very few wrong alignments regardless which cutoff was used for the index (Fig. 4). Moreover, using indexes with small *k* and *w* values also results in higher alignment time as more seeds need to be extracted, the seeds have more matches, and more look-ups need to be done to find the top potential graphs to align to. For example, in this experiment, aligning with k=3 requires a maximum of around 20 000 s in CPU time, whereas it takes a few hundred seconds of CPU time using k=9 (see Supplementary Fig. S2 for all combinations of parameters).

From these results, we can recommend that *k* larger than 3 should be used for alignments against closely related species, and a cutoff of five on the index can be used without losing too many alignments. For full sensitivity, we recommend using a small *k* and not limiting the index to keep all seed hits. However, this will result in a longer alignment time. Supplementary Section S3 contains another experiment for validating the correctness of PanPA.

### 4.2 Aligning unseen sequences from *E.coli*

**Figure 5.**
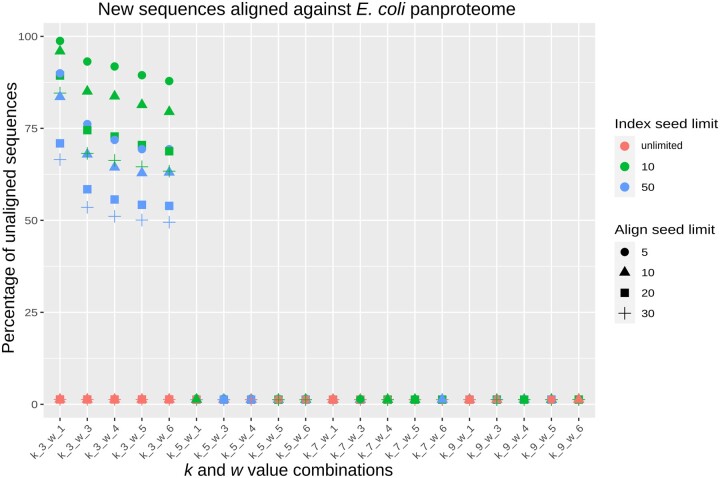
Effect of the different parameters on the number of unaligned sequences when aligning 92 196 unseen *E.coli* sequences. For small *k* values, the majority of sequences were not aligned unless a limit for the index hits size is set (the red marks); if the index hits size is not limited, over 99% of sequences produce an alignment.

Using the same panproteome constructed in the previous experiment, we further downloaded 80 *E.coli* assemblies from RefSeq that were not used in building the panproteome as they were not marked as complete assemblies, and extracted the protein sequences from the corresponding annotation files. After removing redundant sequences, we were left with 92 196 sequences. We used the same Snakemake pipeline as in the previous experiment to align these sequences against the panproteome with the same different parameter combinations. To consider an alignment correct, we require that its sequence identity is above 90%, however, the average alignment score was about 0.998%. We observe again that for small values of *k*, the majority of sequences (between 50% for k=3 and w=6 to 99% for k=3 and w=1) did not produce an alignment (Fig. 5). These results emphasize the conclusion from the previous experiment, that choosing a very small size for the seeds (e.g. k=3) and limiting the index hits size will result in a high number of false positive index hits that; in turn; will result in alignments with a low identity that will be filtered out. When the index hits size is unlimited, PanPA is able to find the correct graphs. However, an unlimited index will result in a much longer alignment time as there is a need to align to more sequences. For example, for k=3,w=1, and unlimited index, it takes PanPA over 80 000 s of CPU time to finish alignments compared to slightly over 1000 s with k=9 and w=1 (Supplementary Fig. S3).

### 4.3 Comparison of PanPA with BWA and GraphAligner using *S.enterica* sequences

One of the major advantages of moving to the amino acid space is the ability to have better alignments between more distant organisms. To test this, we downloaded 1077 *S.enterica* annotated assemblies from RefSeq, extracted all coding regions, and aligned them to the *E.coli* assemblies and graphs that we have already. Both *E.coli* and *S.enterica* belong to the same family *Enterobacteriaceae*, but are from different genera, and hence are expected to be far apart from each other evolutionary to make a good test case for our tool PanPA. In order to compare DNA and protein alignments, we extracted all DNA sequences of coding regions and their corresponding amino acid sequences from the *S.enterica* annotations, obtaining 4 839 981 sequences, which we used to align to the *E.coli* panproteome.

We compared three kinds of alignments here: (i) DNA sequence alignments against the *E.coli* linear reference genome (strain K-12 substrain MG1655) using BWA (Li and Durbin 2010); (ii) DNA sequence alignments using GraphAligner (Rautiainen and Marschall 2020) against the *E.coli* pangenome graph from all 1351 assemblies that we constructed with minigraph (Li *et al.* 2020); and (iii) amino acid sequence alignments using PanPA against the *E.coli* panproteome constructed in the first experiments. Both BWA and GraphAligner were run with default parameters, and PanPA was given an index with k=5, w=5, an index limit of 10, and only aligning each sequence to the top 10 graph hits. The alignments were then filtered based on alignment length and alignment identity, and only alignments with a length of over 50% of the original sequence length and alignment identity of at least 50% were kept.

**Figure 6.**
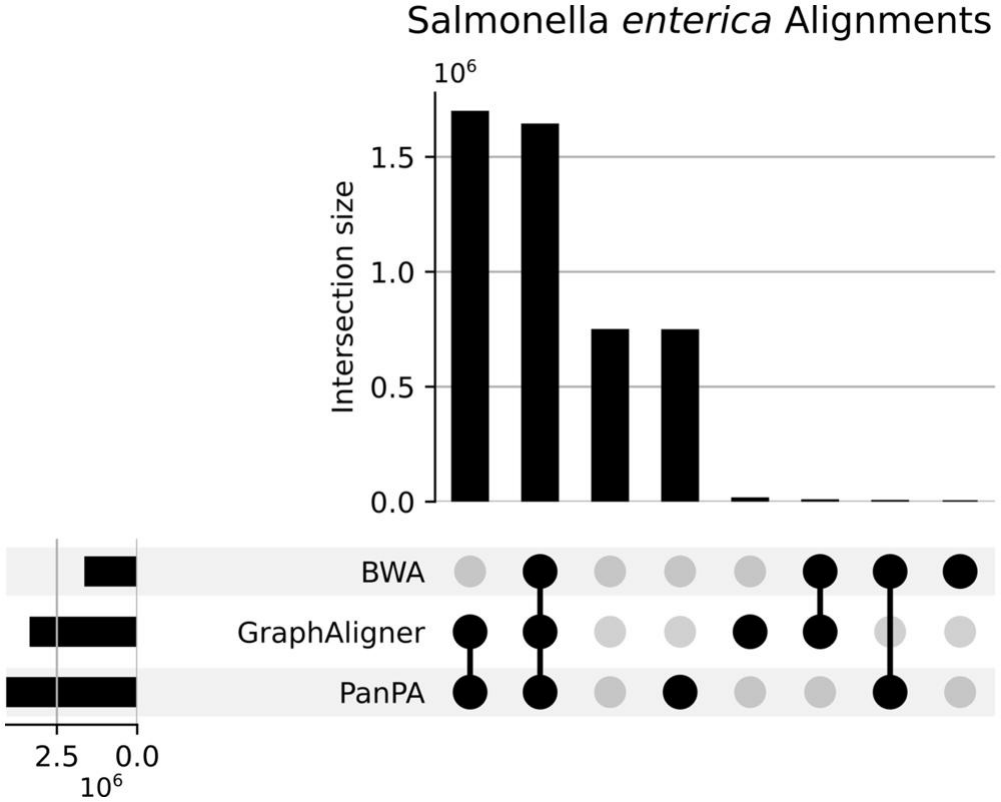
Upset plot of the unique alignments of 4 839 981 sequences from the coding regions of 1074 *S.enterica* assemblies from RefSeq. Alignments with BWA and GraphAligner (DNA), and PanPA (amino acids) against their corresponding *E.coli* counterparts were constructed using the parameters in Supplementary Section S2.

**Figure 7.**
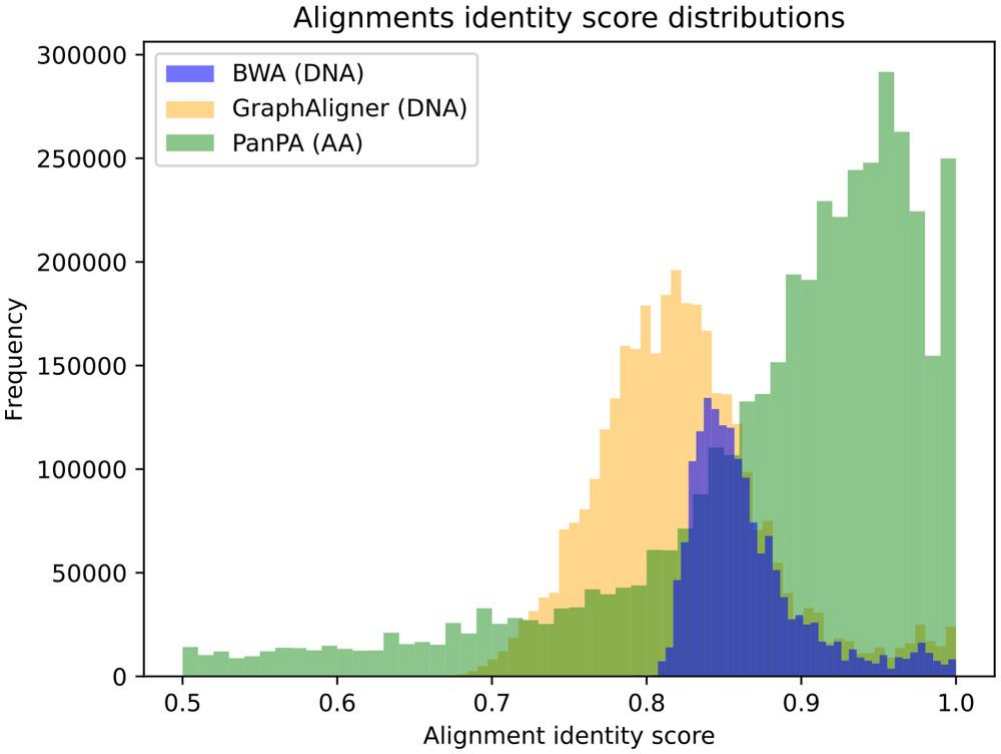
Distribution of identity scores between BWA, GraphAligner, and PanPA from aligning the *S.enterica* sequences. The pique for PanPA is shifted to the right, meaning higher sequence identity, as amino acid sequences align with higher identity compared to nucleotide sequences.

Out of the 4 839 981 sequences, 1 638 936 were successfully aligned by all three aligners, while 1 694 181 could only be aligned by the graph-based methods GraphAligner and PanPA. Strikingly, PanPA could align 744 033 unique sequences that were not aligned by any of the other two aligners (Fig. 6 and Supplementary Table S1). PanPA alignments have higher identity scores, which is to be expected as in the amino acid space the sequence identity is higher for the same two sequences as in the DNA space (Fig. 7). This confirms the advantages of aligning using the amino acid alphabet, which PanPA now enables leveraging for sequence-to-graph alignments. To perform the calculations, PanPA needed around 17 min to build the index, and about 5 h to align the sequences, using 2.3 Gb memory (CPU time 375 515 s), BWA only took around 6 min to run and needed around 900 Mb of memory (CPU time 6818 s), and GraphAligner needed around 20 min to run and used around 700 Mb of memory (CPU time 22 908 s), all of the tools were run with 20 cores.

PanPA did take more time to perform the alignment compared to the other tools. However, PanPA was able to align more sequences, and due to the use of a substitution matrix instead of edit distance in the alignment algorithm, certain algorithmic speeding tricks cannot be used by PanPA. We elaborated more on this point in Section 5.

### 4.4 Aligning *S.enterica* Illumina short reads to the *E.coli* genome, pangenome, and panproteome

PanPA is also able to align DNA sequences to protein graphs by translating each DNA sequence into six different reading frames (three forward and three reverse-complement). This feature can be very helpful for aligning sequencing reads from organisms that do not have a reference genome of the same species or a close enough species to align to.

We downloaded one *S.enterica* Illumina whole genome sequencing short-reads sample (SRR22756191) from NCBI SRA database (Leinonen *et al.* 2011) containing 1 110 471 sequences, the sample is part of PulseNet USA surveillance for food-borne diseases. We aligned the sequences using BWA against the linear reference of *E.coli* that we used in the previous experiment and against the *E.coli* panproteome using PanPA, using the index with k=5, w=3, and no cutoff. In the alignment step, we allowed each sequence to align to up to 20 graphs. We filtered the output retaining alignments with >50% alignment sequence identity and 50% alignment length. BWA was used with default parameters. For PanPA, we chose a relatively small *k*, because we sought higher sensitivity. To match the DNA sequences, it needed to be first translated into six different reading frames and seeds extracted from all frames to find the hits, which can elevate the false positive rate. However, increasing the number of allowed graphs only affected the number of alignments done and the overall run time: alignments that did not pass the identity threshold were not returned. Similar to the experiments above, we advise choosing a smaller seed size with an unlimited index when the user wants higher sensitivity, e.g. in the case of aligning to a more distant organism.

As expected, using a distant linear reference has a major disadvantage: around 65% of the reads could not be aligned with BWA with identity over 50%; and after additional filtering requiring alignment length to be over 50%, only 4.4% were reported retained (Table 1). On the other hand, PanPA was able to produce alignments for 72% of the reads with identity over 50%, and 68% of reads could be aligned over more than 50% of their length. Three lakh fifty-five thousand four hundred and sixty-two sequences were not aligned by either aligner. In this experiment, PanPA needed about 6 h to align the DNA sequences using 10 threads (CPU time 169 004 s), and used about 1.8 Gb of memory. BWA only took 17 s to run with 10 threads (CPU time 162 s).

**Table 1.** Number of *S.enterica* DNA short reads aligned against *E.coli*’s linear reference with BWA and against its panproteome using PanPA.

	Identity >50%	Identity and length >50%
BWA	391 041 (35.2%)	48 937 (4.4%)
PanPA	801 389 (72.2%)	755 009 (68%)

In conclusion, PanPA required over two orders of magnitudes more time compared to bwa. However, looking at the alignment result difference, PanPA was able to align over an order of magnitude more sequences with alignment identity over 50% than bwa.

### 4.5 Using PanPA to display phenotypic traits: a case of antimicrobial resistance in *E.coli*

Certain mutations are associated with bacteria being resistant or susceptible to antibiotics, and this has been a main focus of many researchers, as resistance against antibiotics presents a major threat to public health. We explored the applicability of our tool PanPA to identify such mutations. To this end, we used the Pathosystems Resource Integration Center (Davis *et al.* 2020) database, which contains assemblies and annotations for many antibiotic-resistant and susceptible bacteria. We downloaded ciprofloxacin-resistant and susceptible strains from *E.coli*, which comprised 556 resistant and 1295 susceptible genomes. We extracted two genes, *parC* and *gyrA*, which encode for quinolone, and particularly ciprofloxacin, targets and can carry resistance-associated mutations in *E.coli* (Bagel *et al.* 1999) and translated them to proteins. For each of these two proteins, we were able to extract 1236 susceptible and 309 resistant sequences. We randomly split the sequences into two sets, one containing 10% of the sequences and the other 90% of the sequences. We mixed the 90% sample of both susceptible and resistant together, generated an MSA using mafft, and then a graph for each protein using PanPA().

**Figure 8.**
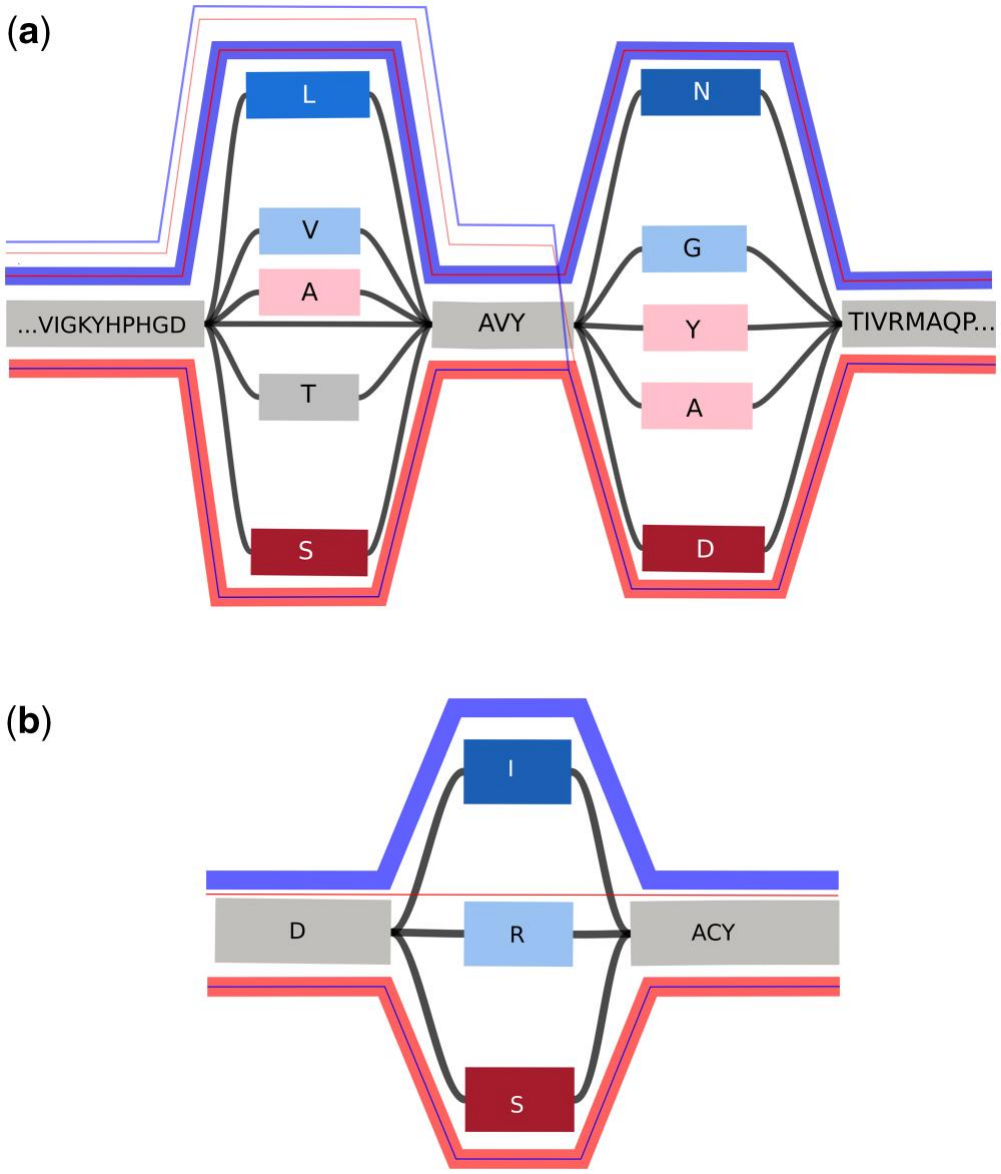
Visualization of parts of the protein graphs for (a) GyrA and (b) ParC using Bandage (Wick *et al.* 2015). Nodes are colored according to the number of resistant/susceptible strains that pass through them, with blue color representing resistance, and with red representing susceptibility; the color intensity corresponds to the number of strains. Additional colored lines show the paths of the aligned 10% sequence that were set aside (45 resistant and 117 susceptible sequences), the color representing the type, and the thickness representing the number of sequences taking that path. A thick blue line of resistant sequences took the blue path passing through the blue nodes, and *vice versa*, a thick red line for susceptible sequences took the red path passing through the red nodes.

In this way, we obtained a graph for each protein containing resistant and susceptible sequences. The variance between the sequences creates bubbles in the graph. Resistance-associated mutations [S83L, D87N in GyrA (Webber *et al.* 2017, Yu *et al.* 2020, Rakici *et al.* 2021), S80I in ParC (Nawaz *et al.* 2015)] are clearly visible in them (Fig. 8). Besides these canonical resistance-associated variants, we observed other potential variants that are present predominantly in resistant strains: alanine, leucine, and valine at Position 83 and alanine, tyrosine, and asparagine at Position 87 of GyrA, as well as arginine at Position 80 of ParC. We aligned the 10% sequence set aside to the graphs using PanPA. Visualizing the corresponding paths (Fig. 8) one can see that the vast majority of the sequences extracted from resistant strains are aligned to the nodes that represent variants associated with resistance, and susceptible sequences aligned to mostly nodes associated with susceptible variants.

### 4.6 Comparing against HMMER

HMMER is a widely used tool for searching for remote homologs in protein databases (Finn *et al.* 2011). HMMER has a high sensitivity, which makes it useful for aligning sequences that have lower similarity due to their large phylogenetic distance from the target. HMMER builds a hidden Markov model for each MSA given, which is then used for aligning a new sequence against the profile.

To compare PanPA’s performance with HMMER, we consider each protein cluster as a separate profile. HMMER can be then used to align new sequences against these profiles and choose the best hits. More formally, we performed two comparative steps between HMMER and PanPA:

1. Building HMM profiles in HMMER, and generating graphs and an index in PanPA, as both are preprocessing steps before doing alignments;
2. HMMER search step and PanPA’s alignment step, as HMMER search also produces alignments.

Again, we used the 44 204 protein clusters of the *E.coli* sample we have from previous experiments. For PanPA, we built a *w*, *k*-minimizer index for the clusters with k=5 and w=3. This step required 17 min and 50 s, and about 1.4 Gb of memory. For building graphs in GFA format for each cluster, PanPA needed 6 min and 37 s using 10 threads. Building HMM profiles from the same alignments with hmmbuild command of HMMER took 2 h, 46 min, and 18 s (using one thread) for all 44 204 clusters. As HMMER runs separately on each MSA, only one thread was used. However, one can use a bash script of Snakemake, e.g. to run several MSAs at the same time on different threads.

For aligning, we extracted a random sample of 10 000 protein sequences from the *S.enterica* sample we used in the previous experiments, and aligned these sequences to graphs or HMMs, respectively. PanPA took 20 min and 57 s (CPU time 959 s) to align all 10 000 sequences back with a minimum alignment identity threshold of 10%. Using 10 threads brought the time down to 7 min and 25 s. PanPA always spends about 5 min loading all the graphs into memory before alignments, which means the more sequence are aligned, the smaller this overhead relative to the total runtime. PanPA used 2.2 Gb of memory, and the number of query sequences does not affect the memory consumption. Therefore, such an example can easily run on any conventional laptop or desktop computer. HMMER took 19 min and 29 s (CPU time 3341 s) to align all 10 000 sequences against the database of HMM profiles constructed previously with the hmmalign command using about 1 Gb of memory and 10 cores. Comparing the results, we found that 9813 query sequences were aligned to the same target cluster by both tools. One hundred and eighty-seven query sequences were aligned by PanPA, but not by HMMER. However, these 187 sequences had very low alignment sequence identity averaging at 25%, which could simply point to random hits, as PanPA was set to report all alignments back even at very low alignment identity.

The major reason for PanPA to be faster than HMMER is the use of the index that guides PanPA on where to align and thus reduces the search space considerably. HMMER aligns each profile to each query sequence, which makes the runtime linear in the number of clusters. PanPA’s ability to run in multiple threads also reduced the alignment time considerably. For example, in this alignment experiment, the actual alignment time for PanPA was 15 min and 47 s using one thread, but only 2 min and 13 s when using 10 threads.

In conclusion, for the preparation step, PanPA needed, in total, around 24 min to generate the index and the graphs, HMMER on the other hand needed around 2 h. For the aligning step, PanPA needed around 7 min (CPU time 117 s) with 10 threads, to align all sequences and HMMER needed around 19 min (CPU time 3341 s) and missed 187 sequences out of the 10 000 query sequences, and both tools reported similar results. More details about time and memory requirements for this experiment are in Supplementary Section S4.

## 5 Discussion

In this article, we present PanPA, a software tool to build and index panproteome graphs, and align sequences to them. In our method, instead of building one big graph that represents all samples of a population, we build many local graphs, where each local graph represents one protein or a group of related proteins.

We demonstrate that PanPA produces correct alignments when aligning a sample of *E.coli* protein sequences back to an *E.coli* panproteome produced from assemblies from a public database.

We also show that moving into amino acid space can increase both the number of aligned sequences and the alignment identity score when comparing phylogenetically distant organisms as exemplified by aligning *S.enterica* proteins against the panproteome constructed from *E.coli* assemblies. PanPA can also capture a much higher number of hits that would have been otherwise lost when using a distant reference. We argue that aligning over longer phylogenetic distances is important, especially when trying to study organisms that are not well-researched, do not have a standard reference, and where a particular clade is only scarcely sequenced. In these cases, one can use a distant organism panproteome to produce better alignments and comparison, maybe advancing one step toward annotation using a remote reference.

Additionally, we demonstrate the utility of PanPA for the discovery of genetic mechanisms of phenotypic traits, such as antimicrobial drug resistance.

We also show that PanPA’s computational resources are reasonable, especially in terms of memory consumption. It can easily be used on any modern laptop or desktop machine without the need of accessing a high-performance computational cluster. Moreover, as PanPA can be parallelized, if the user has access to a computational node with more CPUs, this can make the alignment much faster. However, PanPA is still slower than other linear aligners (e.g. BWA) or graph aligners (e.g. GraphAligner). This stems from the fact that PanPA builds a complete DP table for the alignments and fills all the cells, and it uses different substitution matrices for scoring and not edit distance, which prevents PanPA from applying tricks like bounded edit distance (Ukkonen 1985) or the fast bit-vector algorithm for string matching (Myers 1999). The latter algorithm was also extended to graphs (Rautiainen *et al.* 2019). Therefore, PanPA’s performance bottleneck is not the number of graphs in the panproteome, but how big these graphs are or how sparse their corresponding MSAs are. In Supplementary Section S5, we show that PanPA can still handle very sparse MSA, albeit slower. Therefore, PanPA does still perform well on real datasets, and with its low memory usage, it can run on local machines or small computational nodes, where more CPUs can be used to speed up the alignment step.

## Supplementary Material

vbad167_Supplementary_Data

## Data Availability

The source code and list of accession numbers of samples used is available at https://github.com/fawaz-dabbaghieh/PanPA.
