# Supplementary material for: PanPA: generation and alignment of panproteome graphs
Source: Bioinform Adv. 2023 Nov 24;3(1):vbad167. doi: 10.1093/bioadv/vbad167 (PMC10748787; doi:10.1093/bioadv/vbad167)
Supplement: vbad167_Supplementary_Data [file vbad167_supplementary_data.pdf]

Supplementary Material
`PanPA`: generation and alignment of pan-proteome graphs

November 20, 2023

Intersection	Number of alignments ≥ 50% identity	Number of alignments ≥ 70% identity
Not Aligned	744,964	1,012,744
BWA	1	1
BWA - GraphAligner	4,084	4,090
BWA - PanPA	1,294	1,294
GraphAligner	12,488	52,357
Graphaligner - PanPA	1,694,181	1,643,479
PanPA	744,033	487,086
BWA - GraphAligner - PanPA	1,638,936	1,638,930

Supplementary Table. 1: Intersection of unique alignments of 4,839,981 sequences representing the annotations from Salmonella *enterica* assemblies from RefSeq, against E. *coli* linear reference, pangenome, and panproteome using BWA, GraphAligner, and PanPA respectively.

Aligner	BWA	GraphAligner	PanPA
**Num. alignments**	2,699,361	26,009,077	8,684,414
**Num. filtered alignment 50% length**	1,645,224	4,399,906	7,897,707
**Num. filtered alignments 50% id**	1,645,224	4,399,906	7,897,707
**Num. filtered alignments 70% id**	1,645,222	4,384,913	5,273,200

Supplementary Table. 2: Number of alignments from the 4,839,981 sequences from Salmonella *enterica* annotations using BWA, GraphAligner and PanPA. We see that GraphAligner produced the most alignments. However, after filtering for an alignment length of at least 50% of the original sequence size, the number of alignments drops drastically. For PanPA, most of the alignments were long enough and only a small number got filtered.

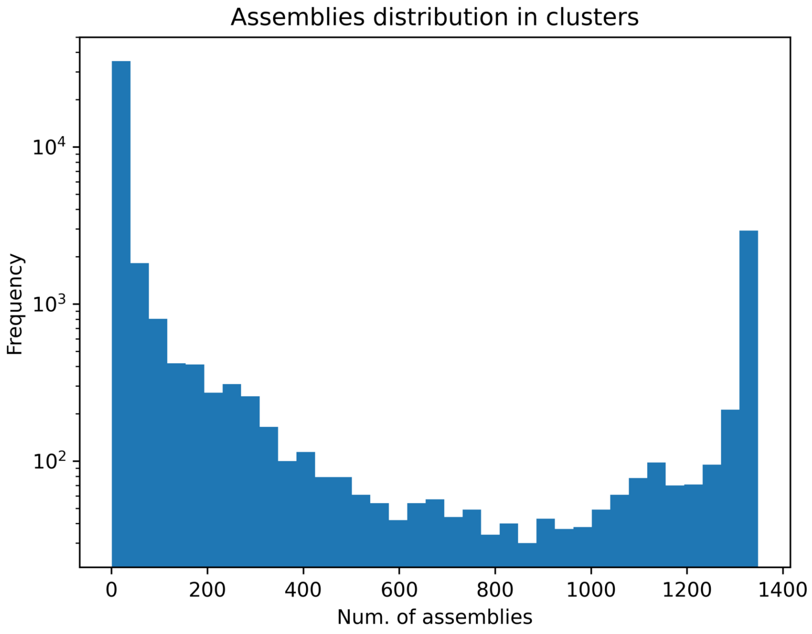

Supplementary Figure. 1: Histogram of number of different strains in each cluster, the typical U-shape cluster where the left peak represent the unique clusters, and the right peak represent the core genes cluster

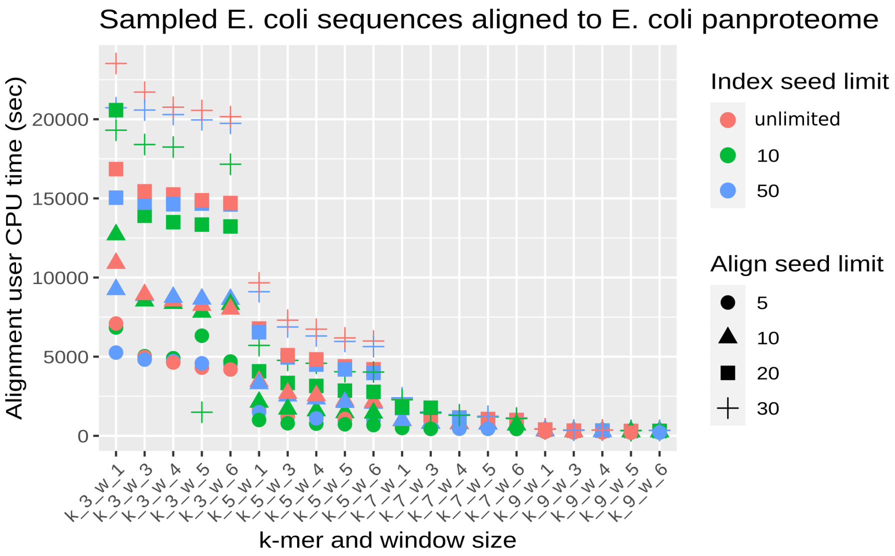

Supplementary Figure. 2: k and w sizes against alignment time for the sampled *E. coli* sequences. We can see the indexes with smaller values for k and w will have higher alignment time.

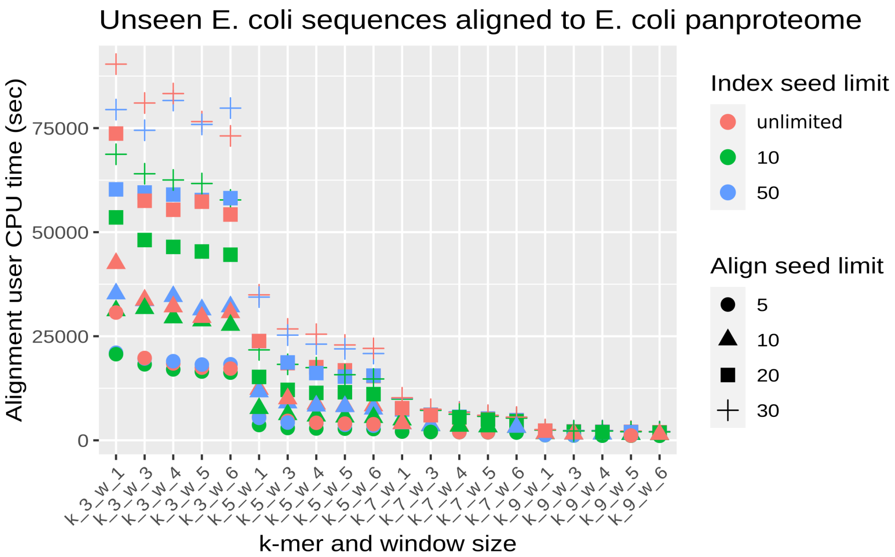

Supplementary Figure. 3: k and w sizes against alignment time for the unseen *E. coli* sequences aligned against the panproteome. We see that for small values of k the time is much higher, especially when the index is unlimited.

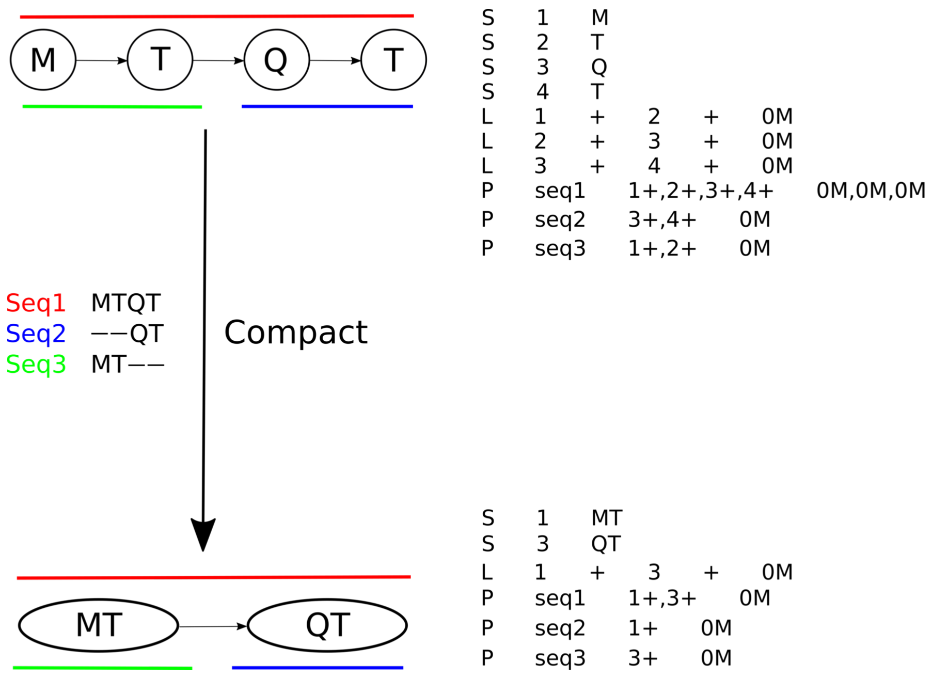

Supplementary Figure. 4: An example of constructing a graph from an MSA with 3 sequences $MTQT$, QT, and MT. The top left graph is the uncompacted graph, where each column in the MSA produced one node, the red line is the path for Sequence 1, the blue for sequence 2, and the green for sequence 3, to the right of the graph is the GFA format that would produce that graph with these paths for each sequence. After compacting, instead of ending up with only one node as we have a path graph, we end up with two nodes, because in the GFA format, a path is represented with nodes, and if we compact all the nodes, we will not be able to represent Sequence 2 and 3. Therefore, nodes 1 and 2 get merged, and 2 and 3 are merged

1 Sequences Random Selection

The 32,289 sequences from the *E. coli* panproteome that were chosen for testing were chosen at random using a script that can be found on `PanPA`'s repository, it takes two arguments as input, both integers from 0 to 100 representing the percentage of different protein clusters to choose at random, and how many sequences to choose from each MSA. We gave the script the inputs 10 and 5, which then chooses at random 10% of the protein clusters, and then from each cluster chooses 5% of the sequences at random. Because we know from which cluster each sequence belongs to, we can calculate the number of matches after doing the alignments.

2 Frameshift-Aware DNA to Protein Alignment

The main idea behind this frameshift-aware alignment is that we are basically aligning the 3 different reading frames against the amino acid sequence using one DP table and allowing penalized switches between the different frames when calculating the scores.
Looking at table 3 that represents a filled DP table of aligning the DNA sequence “CCTCCTGACCCACCAA” against the amino acid sequence “PPTHQ”, where the DNA sequence is represented in the rows and the amino acid sequence is represented in the columns. At each row i, the codon representing that iteration will be the subsequence $[i-2, i]$, i.e. we will start the aligning at the 3rd DNA letter (in this example, T) which will represent the codon “CCT” which gives the amino acid “P”, which matches with the first letter of the amino acid sequence, that's why we have the score 2 between “T” and “P” in the table. Therefore, when we go to the next row, at the DNA letter “C” after “T”, this will represent the codon “CTC” which translates to “L” which does not match with any of the amino acid letters, which results in 0's across that row.
To calculate the score of a cell in the DP tale, we use equation 1, which is a modified Smith-Waterman algorithm, where at each row, when we want to look at the previous codon in the same frame, we need to basically do an $i-3$ jump, every time we jump 3 DNA letters, we jump one codon in the same frame. For example, when we are at the DNA letter “T” before the red letter “G” in the table, this would represent the codon “CCT” which translates to “P” that matches both the first and second “P” in the amino acid sequence, and we get the score 4 because two codons matched now, i.e. the score 4 at row 8 and column 2, came from the score 2 at row 5 and column 1 plus 2 for a match between the codon and the amino acid.
Moreover, in this modified algorithm, when aligning a DNA sequence to an amino acid sequence, we add to the equation two other types of diagonals when calculating a cell at (i, j):

1. an $i-4, j-1$ jump, which describes an insertion frameshift, i.e. the DNA sequence has an extra nucleotide that introduced a frameshift, and this would change the current alignment to the previous frame.

2. an $i-2, j-1$ jump, which describes a deletion frameshift, i.e. the DNA sequence has one nucleotide deleted that caused the frameshift

For these frameshifting jumps, we introduce the frameshift penalty σ. For example, after filling the table, when we trace back from the bottom right corner with score 7, which is the highest score in this table, we first go from score 7 to 5, matching the codon "CAA" with "Q", then from score 5 to 3 matching the codon "ACC" matching "H", but due to the insertion "G" in the DNA sequence, the score 3 actually came from the highlighted score 4 but with a frameshift penalty, which is still better than staying in the same frame, and this -4 jump basically matches "GACC" against the "T". Then from the highlighted score 4 we jump to 2 which matches the codon "CCT" against "P", then 2 came from 0 and matches "CCT" against "P". Therefore, in this formulation, we were able to completely align the DNA sequence against the amino acid sequence regardless of the insertion in the DNA sequence.
In `PanPA`, the CIGAR string produced will be in the DNA space and for this alignment would be 6=1I9=, where "=" means a match and "I" means an insertion.

$$score(i,j) = \max \begin{cases} H_{i-3,j-1} + sub(trans(N[i,i+2]), M[j-1]) \\ H_{i-3,j} + \Delta \\ H_{i,j-1} + \Delta \\ H_{i-4,j-1} + \sigma \\ H_{i-2,j-1} + \sigma \\ 0 \end{cases} \tag{1}$$

3 GyrA protein with introduced variants

To further test the robustness of `PanPA`'s alignments and make sure it is doing correct alignments, we took the graph representing the MS of the protein GyrA used in the Results Section 4.5 and aligned the 1392 protein sequences of both susceptible and resistant strains of GyrA protein back to the graph. With each iteration, we added random errors to the sequence, i.e. we replaced some amino acids with random amino acids. For each alignment run, we compared the path that the alignment took with the path that the original sequence created when the GFA was constructed, we also looked at the alignment identity, as we expect, when the sequence doesn't have any errors, it should align with 100% identity, and the more errors we introduce, the lower the alignment identity gets.
Looking at table 4 we see that when there are no errors, all alignments match exactly the original paths of that sequence, with an alignment identity of 1 (i.e. 100% aligned positions), as expected. The more errors we added, the more the alignment diverged from the original path. However, we see that the average alignment identity is consistent with the amount of error introduced, moreover, we see that most of the path is still covered in the new alignment, but this results from the fact that in the MSA there were many stretches of conserved sequences

		**P**	**P**	**T**	**H**	**Q**
	0	0	0	0	0	0
	0	0	0	0	0	0
	0	0	0	0	0	0
**C**	0	0	0	0	0	0
**C**	0	0	0	0	0	0
**T**	0	2	2	1	0	0
**C**	0	0	0	0	0	0
**C**	0	0	1	1	0	0
**T**	0	2	4	3	2	1
G	0	0	1	1	0	0
**A**	0	0	1	3	2	1
**C**	0	1	3	4	2	1
**C**	0	0	1	3	2	1
**C**	0	2	2	2	3	2
**A**	0	2	3	3	4	3
**C**	0	0	1	2	5	4
**C**	0	1	2	4	3	3
**A**	0	2	4	3	3	4
**A**	0	0	1	2	4	7

Supplementary Table. 3: Frameshift aware alignment of the DNA sequence **CCTCCTGACCCACCAA** against the amino acid sequence **PPTHQ**, where the nucleotide G in red is an insertion, we see that when filling the DP table, we can account for that frame shift if we follow the cells in yellow, and still end with a full alignment. In this formulation, we start filling the table from the 3rd nucleotide (T) which represents the codon (CCT), so each row, represents the codon of the nucleotide and the two nucleotides before it. Therefore, a diagonal jump of -3 is the same as a diagonal jump of -1 in amino acid space

which results in one node, and when errors are introduced in the sequence that would align to that node, it would still align to the node but with mismatches.

**Number of sequences**	**Percentage of error introduced**	**Matching path**	**Mismatching paths**	**Average alignment identity**	**Average path coverage**
1392	0	1392	0	1	1
1392	5	265	1127	0.952	0.988
1392	10	50	1342	0.905	0.98
1392	15	12	1380	0.857	0.971
1392	20	2	1390	0.809	0.962
1392	25	0	1392	0.762	0.952
1392	30	0	1392	0.715	0.94
1392	35	0	1392	0.667	0.931
1392	40	0	1392	0.62	0.919
1392	45	0	1392	0.575	0.901
1392	50	0	1392	0.529	0.889

Supplementary Table. 4: Inserting random SNPs to the Gyra sequences before aligning back to the graph constructed from the MSA of the same query sequences. We see that the Average alignment identity follows properly the Percentage of errors (or SNPs) introduced, which further indicates that `PanPA` is aligning the sequences properly. Moreover, we see that when there are no errors, the alignment path matches the correct path of that sequence in the graph, and once errors are introduced, the paths started diverging, but only by a few nodes, which makes sense, as the graph is mostly made up of bubbles which where the source and sink nodes of these bubbles are the matching sequences in the MSA, and when the errors introduced effect that part of the graph, the path diverges

4 Comparison against `HMMER`

In section "Results" in subsection "Comparing against HMMER" we compared `PanPA` against `HMMER`, where `PanPA` was able to align the sequences to the same matching graphs that `HMMER` reported in its search, more over, `PanPA` was able to align 187 more sequences than `HMMER`, albeit, having very small alignment identity.
In Table 5 we see the effect of different parameters on `PanPA`, and we see that the identity cutoff does not have much of an effect on the performance time, and this can be of course simply contributed to the fact that the alignment needs to be performed anyway to obtain an alignment identity score and then compared with the cutoff. However, as expected, the number of CPUs provided has a major effect on the alignment time. However, it does not affect the graph loading time, as this is done once linearly at the beginning, and the graphs are

kept in memory to have fast access for the alignment part, and even for the complete *E. coli* panproteome, the memory impact is relatively low.

**Number of sequences**	**Identity cutoff percentage**	**Number of threads**	**Graph limit**	**Aligning time mm:ss**	**total time mm:ss**	**memory**
10,000	40	1	10	15:47	20:14	2.2 Gb
10,000	40	10	10	2:13	7:25	2.2 Gb
10,000	10	1	10	16:09	20:57	2.2 Gb
10,000	10	10	10	2:16	7:02	2.2 Gb

Supplementary Table. 5: The effect of the different parameters on `PanPA`'s performance, we see that the identity cutoff does not affect the alignment time much, and this makes sense, as the alignment will be performed anyway to get an alignment identity score and check whether it is below or above the cutoff.

5 Aligning to Sparse MSAs

To further evaluate and test the limits of `PanPA`, we tried to build a graph and align sequences back to a protein family, we took an MSA from Pfam (PF00006.28) representing the ATP Synthase Alpha/Beta family. Due to the nature of protein families, the MSAs tend to be very sparse as the sequences are evolutionary-related, but in terms of sequence identity, it is rather low. In cases like this, the graph resulting from the MSA tends to also be sparse, i.e. contains many nodes representing small substrings and many edges. For this protein family, the MSA contained 40,339 sequences. It took `PanPA` around 3 seconds to build the GFA, and about 2 minutes to align back a sample of 1,000 sequences of the same MSA back using 1 thread. This is relatively high compared to a more conserved MSA, for example taking the MSA representing the gene Araa from the *E. coli* panproteome which contains 21,657 sequences and it only took `PanPA` about 10 seconds to align a sample of 1,000 sequences back to this graph using 1 thread.
The case of the ATP family can be considered an extreme case, as this MSA is quite sparse with many gaps, and the graph constructed consists of 13,463 nodes with a total concatenated sequence of length 15,303. Therefore, `PanPA` needs to build for each query sequence, a DP table of size of $n \times 15303$ where n is the size of the query sequence. Moreover, the average number of incoming edges has an effect here: in this example for instance, each node had - on average - 5 incoming edges, which means that for calculating each cell in the DP table, `PanPA` needs to follow 5 different paths and calculate the scores before choosing the best one. However, this is an extreme case of an MSA and `PanPA` can still handle such graphs and alignments, albeit slower.

6 *S. enterica* Alignments Parameters

6.1 Alignment comparison of *S. enterica* protein sequences

For aligning the DNA sequences from *S. enterica* against the *E. coli* reference genome, we use BWA with the following parameters:

```
$ bwa mem e_coli_reference_GCF_000005845.2_ASM584v2.
    fasta salmonella_refseqdna.fasta -t 60 >
    salmonella_refseqdna_ecoli_ref_genome_bwa.sam
```

We used GraphAligner for aligning *S. enterica* DNA sequences against *E. coli* pangenome that was built using minigraph with the following parameters:

```
$ GraphAligner -f salmonella_refseq_dna.fasta -g
    e_coli_pangenome.gfa -a
    salmonella_refseqdna_ecoli_pangenome.gaf -x vg --
    threads 60 2> graph_align.log

# for building the pangenome, these commands were used
# this command is the initial one to build a graph
minigraph -xggs -t20 e_coli_reference_GCF_000005845.2
    _ASM584v2.fasta e_coli_reference_GCF_000005845.2
    _ASM584v2.fasta > e_coli_pangenome.gfa

# updating the graph by adding one assembly every step
# assemblies_locations.txt is a list of each E. coli
    assembly to update the graph
$ while read r;do minigraph -xggs -t20
    e_coli_pangenome.gfa $r > tmp && mv tmp
    e_coli_pangenome.gfa;done < assemblies_locations.
    txt
```

PanPAwas used with the following parameters:

```
$ PanPA --log_file salmonella_aa.log align -d
    e_coli_gfa/ --index index_k_5_w_5_seed_lim_10.
    pickle -r salmonella_aa.fasta.gz -o
    salmonella_aa_ecoli_panproteome.gaf --min_id_score
    0.5 --cores 50 2> panpa_time.log
```

6.2 Aligning short reads parameters

For aligning the short reads a sample of *S. enterica* from SRA database with accession number SRR22756191. The following command was used for BWA:

```
$ bwa mem -t 5 reference_ecoli_GCF_000005845/
    SRR22756191.fasta > alignments_SRR22756191.sam
```

For PanPA, the following command was used:

```
$ PanPA --log_file running_new_fs_panpa.log align -d
    index_k_5_w_3_seed_lim_0.pickle -r SRR22756191.
    fasta --dna -c 10 -o
    SRR22756191_ecoli_panproteome_sf_panpa.gaf --
    min_id_score 0.35 --seed_limit 20
```

6.3 Comparison with HMMER parameters

The following parameters were used with PanPAto align the 10,000 sequences of *S. enterica* against the *E. coli* panproteome.

```
$ PanPA --log_file panpa_alignment_10k_10core.log
    align -d e_coli_gfa/ --index
    e_coli_msas_index_k5_w3_no_limit.index -r
    random_10k_sequences.fasta -c 10 -o
    panpa_10k_alignments_0.4min_10core.gaf --
    min_id_score 0.4 --seed_limit 10
```

As for running HMMER, first, we need to convert each MSA into an HMM profile using hmmbuild

```
#!/bin/bash

home_dir=e_coli_hmms/
msa_dir=/e_coli_msa/

start=`date +%s`
for f in $msa_dir/*;
do
        base_fasta=$(basename --suffix .fasta $f)
        hmmbuild $base_fasta.hmm $f
        #echo $base_fasta.hmm
        #echo $f
done
end=`date +%s`
echo it took `expr $end - $start` seconds to run hmmer
     on all clusters > ../hmmer_time.txt

# Compressing all the hmms into one file using
    hmmpress
$ hmmpress all_hmms
# which would produced an indexed file with all hmmer
    profiles

# for search hmmsearch was used
$ hmmsearch --cpu 10 --tblout random_10k_sequences.txt
     -o hmmsearch_10k_output_mt.txt -A
```

```
hmmsearch_10k_alignment.sto all_hmms
random_10_sequences.fasta
```

The table produced by HMMER has the sequence hits against profiles, which we used to match with the alignments produced by PanPA
